# 19-Benzo­yloxy-13,16-*seco*-*ent*-beyeran 13,16-lactone

**DOI:** 10.1107/S1600536810051561

**Published:** 2010-12-15

**Authors:** Jin Cai, Xiaoming Zha

**Affiliations:** a Institute of Pharmaceutical Engineering, School of Chemistry and Chemical Engineering, Southeast University, Nanjing 210096, People’s Republic of China; b Jiangsu Center for Drug Screening, China Pharmaceutical University, Nanjing 210009, People’s Republic of China

## Abstract

The title compound, C_27_H_34_O_5_, a beyerane-type diterpenoid prepared by peroxidation and benzoyl­ation of isosteviol, contains a fused six-membered ring system. The O atoms of the benzoic ester and the lactone are disordered with occupancy ratios of 0.6 (4):0.4 (4) and 0.6 (2):0.4 (2), respectively. Three cyclo­hexane rings have chair conformations, whereas the remaining lactone ring adopts a half-chair conformation.

## Related literature

For the pharmaceutical activity of isosteviol, see: Liu *et al.* (2001 ▶); Braguini *et al.* (2003 ▶); Mizushina *et al.* (2005 ▶); Wong *et al.* (2004) ▶; Xu *et al.* (2007 ▶). For ring conformations, see: Cremer & Pople (1975 ▶). For the synthesis of isosteviol derivatives *via* peroxidation and esterification, see: Chou *et al.* (2008 ▶); Wu *et al.* (2009 ▶); Chen (2010 ▶). 
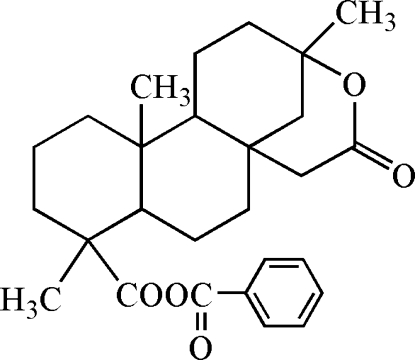


## Experimental

### 

#### Crystal data

C_27_H_34_O_5_
*M* *_r_* = 438.54
Orthorhombic, 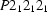

*a* = 7.7425 (16) Å
*b* = 11.871 (2) Å
*c* = 25.306 (5) Å
*V* = 2325.8 (8) Å^3^
*Z* = 4
Mo *K*α radiation
μ = 0.09 mm^−1^
*T* = 298 K
0.48 × 0.46 × 0.43 mm

#### Data collection

Bruker SMART CCD area-detector diffractometer
Absorption correction: multi-scan (*SADABS*; Sheldrick, 2003 ▶) *T*_min_ = 0.960, *T*_max_ = 0.964
12234 measured reflections
2362 independent reflections
1500 reflections with *I* > 2σ(*I*)
*R*_int_ = 0.064

#### Refinement

*R*[*F* ^2^ > 2σ(*F* ^2^)] = 0.051
*wR*(*F* ^2^) = 0.154
*S* = 1.03
2362 reflections
310 parameters
H-atom parameters constrained
Δρ_max_ = 0.18 e Å^−3^
Δρ_min_ = −0.19 e Å^−3^

### 

Data collection: *SMART* (Bruker, 1999 ▶); cell refinement: *SAINT* (Bruker, 1999 ▶); data reduction: *SAINT*; program(s) used to solve structure: *SHELXS97* (Sheldrick, 2008 ▶); program(s) used to refine structure: *SHELXL97* (Sheldrick, 2008 ▶); molecular graphics: *SHELXTL* (Sheldrick, 2008 ▶); software used to prepare material for publication: *SHELXTL* and *PLATON* (Spek, 2009 ▶).

## Supplementary Material

Crystal structure: contains datablocks I, global. DOI: 10.1107/S1600536810051561/rn2071sup1.cif

Structure factors: contains datablocks I. DOI: 10.1107/S1600536810051561/rn2071Isup2.hkl

Additional supplementary materials:  crystallographic information; 3D view; checkCIF report

## Figures and Tables

**Fig. 1.**
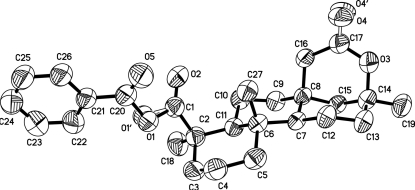
The molecular structure of the title compound with the atom-numbering scheme. H atoms have been omitted. Displacement ellipsoids are drawn at the 30% probability level.

## supplementary crystallographic information

### Comment

Isosteviol, a beyerane-type tetracyclic diterpenoid obtained with stevioside by acid hydrolysis, has a broad spectrum of pharmacological activities against the diseases including hypertension, ischemia-reperfusion injury, and cancer (Wong *et al.*, 2004; Liu *et al.*, 2001; Xu *et al.*, 2007; Mizushina *et al.*, 2005). The title compound was prepared by peroxidation and benzoylation of isosteviol. The molecule contains a fused four-ring system *A/B/C/D*. The *A*/*B* and *B*/*C* junctions are *trans*-fused, and *C*/*D* is *cis*-fused. Six-membered rings *A*, *B* and *C* adopt chair conformations with puckering amplitudes Q = 0.550 (2) / 0.559 (2) / 0.581 (2) Å, θ= 179.6 (2) / 170.4 (3) / 171.8 (3) ° and φ=76 (3) / 76 (2) / 239 (3) °, while the remaining six-membered ring *D* adopts a half-chair conformation with puckering amplitudes Q = 0.517 (6) Å, θ = 136.0 (3)° and φ= 305 (2) °, respectively (Cremer & Pople, 1975). The bond angle of C8—C15—C14 is 111.5 (4)°. The torsion angle of C1—C2—C3—C4 is -71.8 (6)° relates to the *β*-orientation of the benzoyl ester group with respect to the *ent*-kaurane nucleus. The oxygen atoms of the benzoic ester and the lactone are disordered. O1 and O4 are the major components of the disorder. Occupancy ratios of O1/O1' and O4/O4' are 0.6 (4):0.4 (4) and 0.6 (2):0.4 (2), respectively.

### Experimental

Isosteviol was obtained by acid hydrolysis of stevioside with 10% H_2_SO_4_ at 95 °C for 7 h and then recrystallization with ethanol afforded colorless crystals of isosteviol in 80% yield. To a mixture of isosteviol (10 g, 31 mmol) in 150 ml of CH_3_COOH, 90 ml of 30% H_2_O_2_ was added and the mixture reaction was stirred at 60°C for two days. The reaction was cooled to room temperature and poured into ice water. The crude product was filtered and purified by recrystallization with ethanol to give the intermediate 13,16-*seco*-*ent*-beyeran-19-oate 13,16-lactone (8.2 g, 80%) as white crystals.

To a mixture of 13,16-*seco*-*ent*-beyeran-19-oate 13,16-lactone (0.33 g, 0.99 mmol) in 10 ml of CH_2_Cl_2_, pyridine (0.12 ml, 1.5 mmol) and PhCOCl (0.15 ml, 1.3 mmol) was added successively. The mixture reaction was stirred at room temperature for 40 h and washed with diluted HCl, brine and saturated NaHCO_3_ and brine, dried (Na_2_SO_4_) and concentrated under vacuum to give the crude product. Purification of the crude product by a column chromatography (*v:v* petroleum ether: EtOAc= 8:1) afforded the title compound (0.28 g, 65%) as colorless crystals. Crystals of the title compound suitable for X-ray diffraction were obtained by slow evaporation of ethanol solution at room temperature. m.p. 436–437 K; ^1^H NMR (300 MHz, CDCl_3_), δ_H_, p.p.m.: 0.983 (s, 3H), 1.34 (s, 3H), 1.39 (s, 3H), 0.96–2.05 (m, 18H), 2.27–2.32 (d, 1H, J=13.8 Hz), 3.03–3.13 (dd, 1H, J = 18.59, 2.47 Hz), 7.47–7.52 (t, 2H, J=7.7 Hz), 7.62–7.67 (t, 2H, J=14.9 Hz), 7.99–8.02 (m, 1H); ^13^C NMR (75 MHz, CDCl_3_), δ_C_, p.p.m.: 14.4, 18.6, 18.8, 19.6, 28.1, 28.3, 34.9, 37.9, 38.0, 38.5, 38.6, 39.8, 43.6, 45.6, 47.8, 55.9, 57.4, 128.9, 129.2, 130.3, 130.3, 134.4, 162.3, 172.2, 172.6.

### Refinement

The absolute structure could not be established reliably because of insufficient anomalous scattering effects. Therefore, 1404 Friedel opposites were merged. All H atoms were placed in geometrical positions and constrained to ride on their parent atoms with C—H distances in the range 0.96–0.98 Å, and included in the final cycles of refinement using a riding model, with *U_iso_*(H)=1.5*U_eq_*(C) for methyl H and 1.2*U_eq_*(C) for other H atoms. The oxygen atoms of the benzoic ester and the lactone are disordered with occupancy ratios of 0.6 (4): 0.4 (4) and 0.6 (2): 0.4 (2), respectively.

### Crystal data

**Table:**

C_27_H_34_O_5_	*F*(000) = 944
*M**_r_* = 438.54	*D*_x_ = 1.252 Mg m^−^^3^
Orthorhombic, *P*2_1_2_1_2_1_	Mo *K*α radiation, λ = 0.71073 Å
Hall symbol: P 2ac 2ab	Cell parameters from 2537 reflections
*a* = 7.7425 (16) Å	θ = 2.4–20.3°
*b* = 11.871 (2) Å	µ = 0.09 mm^−^^1^
*c* = 25.306 (5) Å	*T* = 298 K
*V* = 2325.8 (8) Å^3^	Prism, colourless
*Z* = 4	0.48 × 0.46 × 0.43 mm

### Data collection

**Table:**

Bruker SMART CCD area-detector diffractometer	2362 independent reflections
Radiation source: fine-focus sealed tube	1500 reflections with *I* > 2σ(*I*)
graphite	*R*_int_ = 0.064
phi and ω scans	θ_max_ = 25.0°, θ_min_ = 1.6°
Absorption correction: multi-scan (*SADABS*; Sheldrick, 2003)	*h* = −5→9
*T*_min_ = 0.960, *T*_max_ = 0.964	*k* = −14→14
12234 measured reflections	*l* = −28→30

### Refinement

**Table:**

Refinement on *F*^2^	Secondary atom site location: difference Fourier map
Least-squares matrix: full	Hydrogen site location: inferred from neighbouring sites
*R*[*F*^2^ > 2σ(*F*^2^)] = 0.051	H-atom parameters constrained
*wR*(*F*^2^) = 0.154	*w* = 1/[σ^2^(*F*_o_^2^) + (0.0594*P*)^2^ + 1.2539*P*] where *P* = (*F*_o_^2^ + 2*F*_c_^2^)/3
*S* = 1.03	(Δ/σ)_max_ = 0.001
2362 reflections	Δρ_max_ = 0.18 e Å^−^^3^
310 parameters	Δρ_min_ = −0.19 e Å^−^^3^
0 restraints	Extinction correction: *SHELXL97* (Sheldrick, 2008), Fc^*^=kFc[1+0.001xFc^2^λ^3^/sin(2θ)]^-1/4^
Primary atom site location: structure-invariant direct methods	Extinction coefficient: 0.023 (3)

### Special details

**Table:**

Geometry. All e.s.d.'s (except the e.s.d. in the dihedral angle between two l.s. planes) are estimated using the full covariance matrix. The cell e.s.d.'s are taken into account individually in the estimation of e.s.d.'s in distances, angles and torsion angles; correlations between e.s.d.'s in cell parameters are only used when they are defined by crystal symmetry. An approximate (isotropic) treatment of cell e.s.d.'s is used for estimating e.s.d.'s involving l.s. planes.
Refinement. Refinement of *F*^2^ against ALL reflections. The weighted *R*-factor *wR* and goodness of fit *S* are based on *F*^2^, conventional *R*-factors *R* are based on *F*, with *F* set to zero for negative *F*^2^. The threshold expression of *F*^2^ > σ(*F*^2^) is used only for calculating *R*-factors(gt) *etc*. and is not relevant to the choice of reflections for refinement. *R*-factors based on *F*^2^ are statistically about twice as large as those based on *F*, and *R*- factors based on ALL data will be even larger.

### Fractional atomic coordinates and isotropic or equivalent isotropic displacement parameters (Å^2^)

**Table:**

	*x*	*y*	*z*	*U*_iso_*/*U*_eq_	Occ. (<1)
O1	0.918 (17)	0.286 (9)	0.376 (4)	0.067 (15)	0.6 (4)
O1'	0.87 (2)	0.259 (13)	0.388 (6)	0.07 (2)	0.4 (4)
O2	0.6430 (6)	0.2441 (3)	0.34276 (13)	0.0755 (11)	
O3	0.5237 (5)	0.1873 (3)	0.05672 (13)	0.0754 (11)	
O4	0.319 (14)	0.287 (9)	0.0949 (8)	0.087 (15)	0.6 (2)
O4'	0.388 (18)	0.324 (8)	0.0979 (18)	0.083 (18)	0.4 (2)
O5	0.8077 (6)	0.4534 (3)	0.37365 (15)	0.0865 (12)	
C1	0.7924 (8)	0.2203 (4)	0.34546 (19)	0.0627 (13)	
C2	0.8863 (7)	0.1252 (4)	0.31854 (18)	0.0609 (13)	
C3	1.0812 (7)	0.1462 (4)	0.3150 (2)	0.0688 (15)	
H3A	1.1236	0.1678	0.3496	0.083*	
H3B	1.1379	0.0763	0.3052	0.083*	
C4	1.1302 (7)	0.2357 (4)	0.2759 (2)	0.0733 (15)	
H4A	1.2551	0.2410	0.2740	0.088*	
H4B	1.0863	0.3078	0.2879	0.088*	
C5	1.0590 (6)	0.2109 (5)	0.22116 (19)	0.0660 (14)	
H5A	1.1146	0.1437	0.2075	0.079*	
H5B	1.0879	0.2729	0.1978	0.079*	
C6	0.8618 (6)	0.1932 (4)	0.22027 (17)	0.0515 (11)	
C7	0.8138 (6)	0.1451 (4)	0.16440 (16)	0.0504 (11)	
H7	0.8949	0.0828	0.1589	0.061*	
C8	0.6345 (6)	0.0916 (4)	0.15766 (16)	0.0507 (11)	
C9	0.5994 (7)	0.0101 (4)	0.20318 (16)	0.0582 (13)	
H9A	0.6739	−0.0551	0.1995	0.070*	
H9B	0.4808	−0.0157	0.2011	0.070*	
C10	0.6299 (7)	0.0636 (4)	0.25735 (17)	0.0585 (13)	
H10A	0.5532	0.1275	0.2619	0.070*	
H10B	0.6049	0.0092	0.2849	0.070*	
C11	0.8170 (7)	0.1023 (4)	0.26179 (17)	0.0533 (12)	
H11	0.8832	0.0365	0.2502	0.064*	
C12	0.8501 (7)	0.2262 (4)	0.11891 (16)	0.0631 (13)	
H12A	0.7699	0.2888	0.1206	0.076*	
H12B	0.9662	0.2560	0.1224	0.076*	
C13	0.8323 (7)	0.1673 (4)	0.06560 (19)	0.0689 (15)	
H13A	0.8412	0.2232	0.0378	0.083*	
H13B	0.9274	0.1149	0.0613	0.083*	
C14	0.6646 (7)	0.1041 (4)	0.05918 (18)	0.0647 (13)	
C15	0.6338 (7)	0.0269 (4)	0.10523 (17)	0.0596 (12)	
H15A	0.7231	−0.0304	0.1060	0.072*	
H15B	0.5234	−0.0105	0.1009	0.072*	
C16	0.4840 (7)	0.1744 (4)	0.15308 (18)	0.0590 (13)	
H16A	0.3798	0.1352	0.1638	0.071*	
H16B	0.5033	0.2343	0.1785	0.071*	
C17	0.4497 (8)	0.2280 (5)	0.1007 (2)	0.0706 (15)	
C18	0.8566 (9)	0.0239 (4)	0.35505 (19)	0.0830 (18)	
H18A	0.9122	−0.0413	0.3405	0.124*	
H18B	0.7349	0.0100	0.3582	0.124*	
H18C	0.9040	0.0397	0.3893	0.124*	
C19	0.6557 (9)	0.0447 (5)	0.00632 (19)	0.0854 (18)	
H19A	0.6759	0.0980	−0.0215	0.128*	
H19B	0.5434	0.0116	0.0020	0.128*	
H19C	0.7420	−0.0133	0.0050	0.128*	
C20	0.8505 (8)	0.3784 (5)	0.4017 (2)	0.0704 (14)	
C21	0.8676 (7)	0.3817 (4)	0.45931 (19)	0.0642 (13)	
C22	0.9247 (8)	0.2913 (5)	0.4883 (2)	0.0811 (17)	
H22	0.9533	0.2242	0.4714	0.097*	
C23	0.9396 (9)	0.3002 (6)	0.5427 (3)	0.0920 (19)	
H23	0.9784	0.2391	0.5624	0.110*	
C24	0.8976 (8)	0.3980 (6)	0.5672 (2)	0.0895 (19)	
H24	0.9076	0.4035	0.6037	0.107*	
C25	0.8411 (9)	0.4880 (6)	0.5388 (2)	0.0886 (18)	
H25	0.8135	0.5547	0.5561	0.106*	
C26	0.8243 (7)	0.4813 (5)	0.4848 (2)	0.0743 (15)	
H26	0.7844	0.5428	0.4656	0.089*	
C27	0.7749 (7)	0.3072 (3)	0.23161 (18)	0.0611 (13)	
H27A	0.8062	0.3602	0.2046	0.092*	
H27B	0.8125	0.3348	0.2654	0.092*	
H27C	0.6518	0.2978	0.2319	0.092*	

### Atomic displacement parameters (Å^2^)

**Table:**

	*U*^11^	*U*^22^	*U*^33^	*U*^12^	*U*^13^	*U*^23^
O1	0.07 (2)	0.064 (16)	0.067 (14)	0.010 (18)	−0.005 (16)	−0.014 (14)
O1'	0.07 (3)	0.06 (2)	0.07 (2)	0.01 (3)	0.00 (2)	−0.01 (2)
O2	0.075 (3)	0.083 (2)	0.069 (2)	0.005 (2)	0.016 (2)	−0.0057 (19)
O3	0.084 (3)	0.085 (2)	0.058 (2)	0.016 (2)	0.005 (2)	0.0064 (19)
O4	0.08 (2)	0.08 (2)	0.097 (6)	0.03 (2)	−0.006 (8)	0.007 (8)
O4'	0.08 (3)	0.08 (2)	0.091 (9)	0.03 (2)	0.004 (12)	0.011 (10)
O5	0.098 (3)	0.083 (3)	0.078 (2)	0.014 (3)	−0.003 (2)	0.007 (2)
C1	0.071 (4)	0.059 (3)	0.057 (3)	0.007 (3)	−0.001 (3)	−0.002 (3)
C2	0.071 (3)	0.053 (3)	0.059 (3)	0.007 (3)	−0.001 (3)	−0.003 (2)
C3	0.069 (4)	0.065 (3)	0.072 (3)	0.014 (3)	−0.011 (3)	−0.013 (3)
C4	0.063 (3)	0.074 (3)	0.084 (4)	−0.001 (3)	−0.001 (3)	−0.017 (3)
C5	0.059 (3)	0.066 (3)	0.073 (3)	−0.008 (3)	0.009 (3)	−0.003 (3)
C6	0.052 (3)	0.047 (3)	0.055 (3)	0.000 (2)	0.008 (2)	−0.002 (2)
C7	0.054 (3)	0.048 (2)	0.049 (2)	0.002 (2)	0.010 (2)	−0.002 (2)
C8	0.053 (3)	0.047 (2)	0.051 (3)	0.001 (2)	0.009 (2)	0.002 (2)
C9	0.063 (3)	0.051 (3)	0.061 (3)	−0.011 (2)	0.006 (2)	0.005 (2)
C10	0.065 (3)	0.055 (3)	0.056 (3)	−0.006 (3)	0.008 (3)	0.012 (2)
C11	0.059 (3)	0.046 (2)	0.055 (3)	0.003 (2)	0.003 (2)	0.000 (2)
C12	0.070 (3)	0.063 (3)	0.056 (3)	−0.008 (3)	0.014 (3)	0.001 (2)
C13	0.075 (4)	0.078 (3)	0.054 (3)	−0.002 (3)	0.016 (3)	−0.001 (3)
C14	0.067 (3)	0.073 (3)	0.054 (3)	0.005 (3)	0.011 (3)	−0.003 (3)
C15	0.061 (3)	0.059 (3)	0.059 (3)	0.005 (3)	0.007 (3)	−0.009 (2)
C16	0.060 (3)	0.064 (3)	0.053 (3)	0.007 (3)	0.009 (2)	0.001 (2)
C17	0.076 (4)	0.073 (4)	0.063 (3)	0.017 (3)	0.007 (3)	0.004 (3)
C18	0.116 (5)	0.065 (3)	0.068 (3)	0.011 (4)	−0.011 (4)	0.011 (3)
C19	0.088 (4)	0.107 (4)	0.061 (3)	0.001 (4)	0.008 (3)	−0.019 (3)
C20	0.071 (4)	0.071 (4)	0.069 (3)	0.011 (3)	−0.004 (3)	−0.015 (3)
C21	0.061 (3)	0.067 (3)	0.065 (3)	0.003 (3)	−0.002 (3)	−0.014 (3)
C22	0.085 (4)	0.080 (4)	0.079 (4)	0.005 (4)	−0.011 (3)	−0.012 (3)
C23	0.091 (5)	0.098 (5)	0.087 (4)	−0.002 (4)	−0.010 (4)	0.004 (4)
C24	0.077 (4)	0.116 (5)	0.076 (4)	−0.001 (4)	0.000 (4)	−0.014 (4)
C25	0.082 (4)	0.096 (4)	0.088 (4)	0.004 (4)	0.003 (4)	−0.031 (4)
C26	0.069 (4)	0.077 (3)	0.076 (4)	0.005 (3)	−0.001 (3)	−0.018 (3)
C27	0.077 (3)	0.045 (3)	0.062 (3)	0.003 (3)	0.007 (3)	0.002 (2)

### Geometric parameters (Å, °)

**Table:**

O1—C20	1.38 (2)	C10—H10B	0.9700
O1—C1	1.47 (9)	C11—H11	0.9800
O1'—C1	1.31 (4)	C12—C13	1.526 (6)
O1'—C20	1.47 (10)	C12—H12A	0.9700
O2—C1	1.193 (6)	C12—H12B	0.9700
O3—C17	1.341 (6)	C13—C14	1.509 (7)
O3—C14	1.473 (6)	C13—H13A	0.9700
O4—C17	1.24 (3)	C13—H13B	0.9700
O4'—C17	1.24 (4)	C14—C15	1.501 (6)
O5—C20	1.185 (6)	C14—C19	1.514 (6)
C1—C2	1.505 (7)	C15—H15A	0.9700
C2—C3	1.532 (8)	C15—H15B	0.9700
C2—C18	1.534 (6)	C16—C17	1.495 (7)
C2—C11	1.557 (6)	C16—H16A	0.9700
C3—C4	1.501 (7)	C16—H16B	0.9700
C3—H3A	0.9700	C18—H18A	0.9600
C3—H3B	0.9700	C18—H18B	0.9600
C4—C5	1.519 (7)	C18—H18C	0.9600
C4—H4A	0.9700	C19—H19A	0.9600
C4—H4B	0.9700	C19—H19B	0.9600
C5—C6	1.541 (6)	C19—H19C	0.9600
C5—H5A	0.9700	C20—C21	1.465 (7)
C5—H5B	0.9700	C21—C22	1.373 (7)
C6—C27	1.538 (6)	C21—C26	1.389 (7)
C6—C11	1.546 (6)	C22—C23	1.386 (8)
C6—C7	1.569 (6)	C22—H22	0.9300
C7—C12	1.526 (6)	C23—C24	1.355 (9)
C7—C8	1.537 (7)	C23—H23	0.9300
C7—H7	0.9800	C24—C25	1.359 (8)
C8—C9	1.528 (6)	C24—H24	0.9300
C8—C16	1.529 (6)	C25—C26	1.375 (7)
C8—C15	1.533 (6)	C25—H25	0.9300
C9—C10	1.529 (6)	C26—H26	0.9300
C9—H9A	0.9700	C27—H27A	0.9600
C9—H9B	0.9700	C27—H27B	0.9600
C10—C11	1.524 (7)	C27—H27C	0.9600
C10—H10A	0.9700		
			
C20—O1—C1	115 (6)	H12A—C12—H12B	108.0
C1—O1'—C20	119 (6)	C14—C13—C12	113.6 (4)
C17—O3—C14	121.5 (4)	C14—C13—H13A	108.8
O2—C1—O1'	113 (6)	C12—C13—H13A	108.8
O2—C1—O1	123 (2)	C14—C13—H13B	108.8
O1'—C1—O1	23 (6)	C12—C13—H13B	108.8
O2—C1—C2	128.4 (5)	H13A—C13—H13B	107.7
O1'—C1—C2	115 (3)	O3—C14—C15	109.0 (4)
O1—C1—C2	108 (2)	O3—C14—C13	108.0 (4)
C1—C2—C3	112.4 (4)	C15—C14—C13	110.9 (4)
C1—C2—C18	104.0 (4)	O3—C14—C19	104.0 (4)
C3—C2—C18	108.1 (5)	C15—C14—C19	113.2 (4)
C1—C2—C11	112.4 (4)	C13—C14—C19	111.5 (5)
C3—C2—C11	108.3 (4)	C14—C15—C8	111.5 (4)
C18—C2—C11	111.5 (4)	C14—C15—H15A	109.3
C4—C3—C2	113.8 (5)	C8—C15—H15A	109.3
C4—C3—H3A	108.8	C14—C15—H15B	109.3
C2—C3—H3A	108.8	C8—C15—H15B	109.3
C4—C3—H3B	108.8	H15A—C15—H15B	108.0
C2—C3—H3B	108.8	C17—C16—C8	118.5 (4)
H3A—C3—H3B	107.7	C17—C16—H16A	107.7
C3—C4—C5	111.8 (4)	C8—C16—H16A	107.7
C3—C4—H4A	109.3	C17—C16—H16B	107.7
C5—C4—H4A	109.3	C8—C16—H16B	107.7
C3—C4—H4B	109.3	H16A—C16—H16B	107.1
C5—C4—H4B	109.3	O4—C17—O4'	32.7 (15)
H4A—C4—H4B	107.9	O4—C17—O3	117.0 (11)
C4—C5—C6	113.5 (4)	O4'—C17—O3	116.7 (14)
C4—C5—H5A	108.9	O4—C17—C16	119 (2)
C6—C5—H5A	108.9	O4'—C17—C16	121 (2)
C4—C5—H5B	108.9	O3—C17—C16	120.4 (5)
C6—C5—H5B	108.9	C2—C18—H18A	109.5
H5A—C5—H5B	107.7	C2—C18—H18B	109.5
C27—C6—C5	108.1 (4)	H18A—C18—H18B	109.5
C27—C6—C11	112.9 (4)	C2—C18—H18C	109.5
C5—C6—C11	107.9 (4)	H18A—C18—H18C	109.5
C27—C6—C7	112.6 (4)	H18B—C18—H18C	109.5
C5—C6—C7	107.3 (4)	C14—C19—H19A	109.5
C11—C6—C7	107.8 (3)	C14—C19—H19B	109.5
C12—C7—C8	110.1 (4)	H19A—C19—H19B	109.5
C12—C7—C6	114.0 (4)	C14—C19—H19C	109.5
C8—C7—C6	117.7 (4)	H19A—C19—H19C	109.5
C12—C7—H7	104.5	H19B—C19—H19C	109.5
C8—C7—H7	104.5	O5—C20—O1	115 (5)
C6—C7—H7	104.5	O5—C20—C21	127.0 (5)
C9—C8—C16	109.2 (4)	O1—C20—C21	117 (4)
C9—C8—C15	109.6 (3)	O5—C20—O1'	128 (4)
C16—C8—C15	104.7 (4)	O1—C20—O1'	24 (2)
C9—C8—C7	109.8 (4)	C21—C20—O1'	105 (6)
C16—C8—C7	115.5 (4)	C22—C21—C26	119.7 (5)
C15—C8—C7	107.8 (4)	C22—C21—C20	122.7 (5)
C8—C9—C10	112.7 (4)	C26—C21—C20	117.6 (5)
C8—C9—H9A	109.1	C21—C22—C23	119.8 (6)
C10—C9—H9A	109.1	C21—C22—H22	120.1
C8—C9—H9B	109.1	C23—C22—H22	120.1
C10—C9—H9B	109.1	C24—C23—C22	120.0 (6)
H9A—C9—H9B	107.8	C24—C23—H23	120.0
C11—C10—C9	109.7 (4)	C22—C23—H23	120.0
C11—C10—H10A	109.7	C23—C24—C25	120.6 (6)
C9—C10—H10A	109.7	C23—C24—H24	119.7
C11—C10—H10B	109.7	C25—C24—H24	119.7
C9—C10—H10B	109.7	C24—C25—C26	120.6 (6)
H10A—C10—H10B	108.2	C24—C25—H25	119.7
C10—C11—C6	112.0 (4)	C26—C25—H25	119.7
C10—C11—C2	116.6 (4)	C25—C26—C21	119.2 (6)
C6—C11—C2	115.3 (4)	C25—C26—H26	120.4
C10—C11—H11	103.6	C21—C26—H26	120.4
C6—C11—H11	103.6	C6—C27—H27A	109.5
C2—C11—H11	103.6	C6—C27—H27B	109.5
C13—C12—C7	111.2 (4)	H27A—C27—H27B	109.5
C13—C12—H12A	109.4	C6—C27—H27C	109.5
C7—C12—H12A	109.4	H27A—C27—H27C	109.5
C13—C12—H12B	109.4	H27B—C27—H27C	109.5
C7—C12—H12B	109.4		
			
C20—O1'—C1—O2	−51 (23)	C18—C2—C11—C10	53.1 (6)
C20—O1'—C1—O1	68 (18)	C1—C2—C11—C6	71.1 (6)
C20—O1'—C1—C2	148 (12)	C3—C2—C11—C6	−53.7 (6)
C20—O1—C1—O2	0(15)	C18—C2—C11—C6	−172.5 (4)
C20—O1—C1—O1'	−73 (11)	C8—C7—C12—C13	−55.7 (5)
C20—O1—C1—C2	178 (8)	C6—C7—C12—C13	169.6 (4)
O2—C1—C2—C3	159.0 (5)	C7—C12—C13—C14	51.1 (6)
O1'—C1—C2—C3	−44 (13)	C17—O3—C14—C15	34.9 (6)
O1—C1—C2—C3	−19 (7)	C17—O3—C14—C13	−85.6 (6)
O2—C1—C2—C18	−84.3 (7)	C17—O3—C14—C19	155.9 (5)
O1'—C1—C2—C18	73 (13)	C12—C13—C14—O3	68.0 (5)
O1—C1—C2—C18	97 (7)	C12—C13—C14—C15	−51.3 (6)
O2—C1—C2—C11	36.5 (7)	C12—C13—C14—C19	−178.4 (4)
O1'—C1—C2—C11	−166 (13)	O3—C14—C15—C8	−61.8 (5)
O1—C1—C2—C11	−142 (7)	C13—C14—C15—C8	56.8 (5)
C1—C2—C3—C4	−71.8 (6)	C19—C14—C15—C8	−177.0 (5)
C18—C2—C3—C4	174.0 (4)	C9—C8—C15—C14	178.9 (4)
C11—C2—C3—C4	53.0 (5)	C16—C8—C15—C14	61.9 (5)
C2—C3—C4—C5	−55.2 (6)	C7—C8—C15—C14	−61.6 (5)
C3—C4—C5—C6	55.2 (6)	C9—C8—C16—C17	−154.6 (4)
C4—C5—C6—C27	69.4 (5)	C15—C8—C16—C17	−37.3 (6)
C4—C5—C6—C11	−53.0 (5)	C7—C8—C16—C17	81.1 (5)
C4—C5—C6—C7	−168.9 (4)	C14—O3—C17—O4	−171 (7)
C27—C6—C7—C12	54.5 (5)	C14—O3—C17—O4'	152 (9)
C5—C6—C7—C12	−64.3 (5)	C14—O3—C17—C16	−11.8 (8)
C11—C6—C7—C12	179.7 (4)	C8—C16—C17—O4	173 (7)
C27—C6—C7—C8	−76.6 (5)	C8—C16—C17—O4'	−149 (9)
C5—C6—C7—C8	164.5 (4)	C8—C16—C17—O3	13.9 (8)
C11—C6—C7—C8	48.5 (5)	C1—O1—C20—O5	−70 (13)
C12—C7—C8—C9	179.7 (4)	C1—O1—C20—C21	121 (7)
C6—C7—C8—C9	−47.4 (5)	C1—O1—C20—O1'	58 (11)
C12—C7—C8—C16	−56.3 (5)	C1—O1'—C20—O5	−18 (26)
C6—C7—C8—C16	76.6 (5)	C1—O1'—C20—O1	−83 (19)
C12—C7—C8—C15	60.4 (4)	C1—O1'—C20—C21	152 (17)
C6—C7—C8—C15	−166.7 (4)	O5—C20—C21—C22	−177.4 (6)
C16—C8—C9—C10	−76.2 (5)	O1—C20—C21—C22	−9(8)
C15—C8—C9—C10	169.7 (4)	O1'—C20—C21—C22	12 (9)
C7—C8—C9—C10	51.4 (5)	O5—C20—C21—C26	2.3 (10)
C8—C9—C10—C11	−60.0 (5)	O1—C20—C21—C26	170 (8)
C9—C10—C11—C6	61.9 (5)	O1'—C20—C21—C26	−168 (9)
C9—C10—C11—C2	−162.2 (4)	C26—C21—C22—C23	−0.4 (9)
C27—C6—C11—C10	70.7 (5)	C20—C21—C22—C23	179.4 (6)
C5—C6—C11—C10	−169.9 (4)	C21—C22—C23—C24	0.1 (10)
C7—C6—C11—C10	−54.3 (5)	C22—C23—C24—C25	−0.1 (10)
C27—C6—C11—C2	−65.8 (6)	C23—C24—C25—C26	0.4 (10)
C5—C6—C11—C2	53.6 (5)	C24—C25—C26—C21	−0.7 (10)
C7—C6—C11—C2	169.2 (4)	C22—C21—C26—C25	0.6 (9)
C1—C2—C11—C10	−63.3 (6)	C20—C21—C26—C25	−179.1 (6)
C3—C2—C11—C10	171.9 (4)		
